# Performance of the Cardiovascular Point of Care Ultrasound (POCUS) Exam by Internal Medicine Residents

**DOI:** 10.24908/pocusj.v10i01.17791

**Published:** 2025-04-15

**Authors:** Christopher Chew, Katherine Lang, Manuel De La Rosa, Amanda K. Bertram, Ariella Apfel Stein, Apurva Sharma, Timothy M. Niessen, Brian T. Garibaldi

**Affiliations:** 1 Department of Medicine, Johns Hopkins University School of Medicine, Baltimore, MD,USA; 2 Hospitalist Program, Division of General Internal Medicine, Johns Hopkins University School of Medicine, Baltimore, MD,USA; 3 Division of General Internal Medicine, Johns Hopkins University School of Medicine, Baltimore, MD,USA; 4 Division of Cardiology, Icahn School of Medicine at Mount Sinai, New York City, NY, USA; 5 Center for Bedside Medicine, Northwestern University Feinberg School of Medicine, Chicago, IL, USA

**Keywords:** cardiac POCUS, bedside ultrasound, internship and residency, physical examination, cardiology

## Abstract

**Background:**

Few studies have examined internal medicine residents' performance using cardiovascular point of care ultrasound (POCUS).

**Methods:**

From 2019 to 2022, first-year residents from two academic medical centers in Baltimore participated in the Assessment of Examination and Communication Skills (APECS). Interns examined a single patient with aortic insufficiency and were assessed on physical exam and POCUS technique, identifying physical exam and POCUS findings, generating a differential diagnosis, clinical judgment, and maintaining patient welfare. Spearman's correlation test was used to describe associations between clinical domains. Preceptor comments were examined to identify common errors in physical exam and POCUS exam technique and in identifying correct findings.

**Results:**

Fifty-three first-year residents (interns) performed a cardiovascular POCUS exam. Of these, 44 (83%) scored either “unsatisfactory” or “borderline” on their POCUS technique with a mean score of 29.5 (out of 100). Seventeen (32%) interns were able to correctly obtain a parasternal-long axis (PLAX) view with only 26 (52%) attempting an apical four-chamber (AP4) or subcostal (SUBC) view. Of the 11 participants who correctly obtained both PLAX and parasternal-short views (PSAX), 10 were able to properly identify a normal ejection fraction and the absence of a pericardial effusion. POCUS technique was statistically significantly associated with physical exam technique, identifying the correct POCUS findings, and generating a correct differential diagnosis (r=0.46, p<0.01; r=0.41, p=<0.01; r=0.60, p=<0.01, respectively).

**Conclusion:**

Internal medicine interns showed variable skill in performing and interpreting a cardiovascular POCUS exam. Further emphasis on teaching cardiovascular POCUS skills would likely increase ability to identify relevant cardiovascular findings and improve patient care.

## Introduction

Imaging technology is often cited as a reason for the decline in physical examination skills. Within cardiology, the blame rests squarely on the increasing prevalence of echocardiography [1–5]. By extending beyond the physical senses of inspection, palpation, and auscultation, formal echocardiograms allow for direct visualization of cardiac structures and improve diagnostic accuracy [6,7]. While traditional echocardiograms have proven themselves essential, the cost of imaging and delay in obtaining formal results often hinder the abilities of clinicians to assess and manage patients in real time [1,7,8]. With the advent of hand-held ultrasound devices that are easily used at the bedside (point of care ultrasound, POCUS), imaging technology has become part of the bedside toolkit alongside the stethoscope [6,7].

POCUS first emerged in the field of Emergency Medicine in 1990 [9]. It is defined as a limited ultrasound examination performed by a clinician at the bedside to answer focused clinical questions [10]. Improvements in the portability, accessibility, cost, and image quality of POCUS devices have made them commonplace. POCUS is now widely used within internal medicine, aiding in timely diagnosis and management [11]. When used to augment a physical exam, POCUS reduces duration of hospital stay, avoids expensive diagnostic imaging, and decreases procedure-related complications [12].

Despite its growing use, the accuracy of a POCUS exam is inextricably tied to the skills of the individual operator, and as a result, the diagnostic yield can vary greatly between clinicians [6,13–15]. There are few studies quantifying the number of POCUS exams needed to ensure an operator achieves competence in obtaining and interpreting images [16]. The paucity and heterogeneity of formalized POCUS curricula and lack of standardized assessments within internal medicine training programs portends the possibility of POCUS misuse, potentiating misdiagnosis and patient harm [11].

Cardiovascular complaints are among the most common causes for hospital admission [17–19]. Since the cardiovascular POCUS exam incorporates multiple different views, it is arguably the exam that is most affected by variability in POCUS training [6,13,15]. The purposes of this study were to determine the overall proficiency of internal medicine interns in performing cardiovascular POCUS, assess how POCUS skills relate to traditional cardiovascular physical exam skills, and examine how POCUS skills impact patient management. We hope to provide a foundation upon which formalized cardiac POCUS curricula can be built so that trainees can utilize POCUS to augment their physical exam skills and improve patient care.

## Methods

Between 2019 and 2022, first-year internal medicine residents (interns) from two academic medical centers in Baltimore, Maryland participated in the Assessment of Examination and Communication Skills (APECS). APECS is a simulated clinical experience in which residents examine real patients with established diagnoses while being evaluated by faculty preceptors [20]. Participants rotated through five stations where they completed a targeted history and/or physical exam (cardiovascular, abdominal, neurological, respiratory, or musculoskeletal) on up to seven patients. Some APECS sessions included an integrated station that included a cardiac physical exam followed by a cardiovascular POCUS exam. Each station was staffed by up to two faculty preceptors who examined the patient the morning of the assessment. They agreed upon the findings that were present and what the expected clinical management would be for each patient. Participants were randomly assigned to attend an APECS session during their intern year (from September through June).

A subset of interns at the integrated cardiovascular physical and POCUS station examined the same male patient with severe aortic insufficiency. Interns who performed a cardiovascular and POCUS exam on a different volunteer patient or who encountered the same patient at a physical exam-only station were excluded. Prior to the encounter, interns were provided with the patient's chief concern—“shortness of breath.” Interns had six minutes to perform a cardiovascular physical exam, and four minutes to report their findings, construct a differential diagnosis, and recommend further diagnostic testing and management. Following the physical exam, interns were instructed to perform a focused cardiovascular POCUS exam in six minutes. During this exam, they were instructed to obtain all five cardiac POCUS views (parasternal long axis [PLAX], parasternal short axis [PSAX], apical four-chamber [AP4], subcostal [SUBC], and inferior vena cava [IVC]). Afterwards, interns were allotted four minutes to report the ultrasound findings, refine their differential diagnosis, and recommend further diagnostic testing or treatment options based on the differential. Following each session, interns were given hands-on feedback to improve their cardiovascular physical exam and ultrasound skills.

Each intern was assessed using a standardized APECS Scoresheet designed to evaluate 1) physical exam technique, 2) identification of physical exam findings, 3) ultrasound technique, 4) identification of ultrasound findings, 5) differential diagnosis, 6) clinical judgment and treatment plan, and 7) ability to maintain patient welfare. Interns received a numerical score (0-2) for each category (Needs Improvement/Unsatisfactory = 0, Borderline = 1, Satisfactory = 2). Preceptors were required to provide written comments for all interns who scored below “satisfactory” for a given clinical domain. Some interns had two faculty preceptors present during the assessment, while others had only one observer. To account for this difference, intern scores were converted to a percentage score (0-100) for each domain. To do this, the total score for each domain was divided by the maximum possible score and multiplied by 100.

In addition to the APECS Scoresheet, interns were also evaluated using an additional POCUS Scoresheet for their POCUS exam at the cardiovascular station. This rubric provided further nuanced data regarding participants' ability to obtain the correct depth, gain, orientation, and structures for all five ultrasound views (PLAX, PSAX, AP4, SUBC, and IVC). Interns received a score (0-2) for each category (Did not attempt = 0, Incorrect view = 1, Correct view = 2).

Descriptive statistics were calculated for each skill domain. Correlations between domains were calculated using Spearman's correlation coefficients. Comments were reviewed by two authors (KL and CC) and collated to further understand common errors in POCUS technique and missed POCUS findings. Participants who did not have both an APECS Scoresheet and a POCUS Scoresheet were excluded from analysis. All data collection and recruitment protocols were reviewed and approved by the Johns Hopkins Medicine Institutional Review Board (Protocol IRB00115500). Some results from the cardiovascular physical exam portion of the assessment have been previously reported [21].

## Results

Between June 2019 and September 2022, 63 interns performed a cardiovascular physical exam and POCUS exam on the same patient with severe aortic insufficiency across 7 different APECS days. Of these, 53 interns had both an APECS and POCUS Scoresheet and were included in the analysis. Six faculty preceptors participated (two from pulmonary and critical care, one from emergency medicine, three from general internal medicine). All faculty routinely used bedside POCUS to teach clinical skills. Three faculty were credentialed to use POCUS in their clinical practice. The three remaining faculty were paired with a credentialed faculty for each session.

During the calibration process prior to each APECS session, faculty agreed that the patient had an early decrescendo diastolic murmur (in seven out of the seven sessions), systolic murmur (in five out of the seven sessions), displaced point of maximal impulse (in six out of the seven sessions), and “water-hammer” pulses (in five out of the seven sessions). On POCUS exam, faculty preceptors agreed in all seven sessions that the patient had a normal ejection fraction, no pericardial effusion, an IVC with respiratory variation, left ventricular hypertrophy, and a dilated aortic root. Color Doppler evaluation was not expected or required to receive a satisfactory score. Twenty-four interns were evaluated by two faculty preceptors; 29 interns were evaluated by only one faculty preceptor.

Of the 53 interns, 44 (83%) scored either “unsatisfactory” or “borderline” on their cardiac ultrasound technique with a total mean score of 29.5 (SD 28.1; Table 1). Interns performed similarly in their ability to identify the correct ultrasound findings, with 41 (77%) interns scoring either “unsatisfactory” or “borderline” with a total mean score of 28.3 (SD 31.6). The mean scores for identifying the correct differential diagnosis, demonstrating clinical judgement, and maintaining patient welfare were 41.0 (SD 47.6), 73.6 (SD 35.5), and 90.5 (SD 24.1), respectively.

**Table 1.** Mean score and standard deviation for interns' performance in each clinical domain.

Clinical Domain	Mean Score	Standard Deviation
**Physical Exam Technique**	54.2	36.1
**Identifying Physical Exam Finding**	27.4	31.1
**POCUS Exam Technique**	29.5	28.1
**Identifying POCUS Finding**	28.3	31.6
**Differential Diagnosis**	41.0	47.6
**Clinical Judgement**	73.6	35.5
**Maintaining Patient Welfare**	90.5	24.1

Only 11 (21%) interns were able to properly capture the correct anatomy in both the PLAX (mitral and aortic valve) and PSAX view (papillary muscles; Table 2). Twenty-six (52%) interns attempted an AP4 or SUBC, while only 11 (21%) interns were able to obtain a satisfactory view of either one. The most common view attempted was the PLAX (100%); 39 interns (74%) were unable to obtain more than one view. The most common causes for “unsatisfactory” or “borderline” technique were improper depth (30 interns, 57%), improper gain (22 interns, 42%), poor orientation of the probe marker to the appropriate physical landmarks (33 interns, 63%), incorrect visualization of the IVC (47 interns, 89%), and failure to attempt any views past the parasternal long and parasternal short view (27 interns, 51%).

**Table 2.** Details the number of interns who attempted each cardiac point of care ultrasound (POCUS) view and the number who correctly obtained each view.

POCUS Views	Attempted View	Correctly Obtained View n (% of all interns)
**PLAX**	53	17 (32%)
**PSAX**	39	14 (26%)
**AP4**	26	9 (17%)
**SUBC**	20	7 (13%)
**IVC**	20	6 (11%)

Of the nine interns who obtained all “satisfactory” scores in ultrasound technique, all were able to correctly identify a normal ejection fraction and the absence of a pericardial effusion. Eight of the nine interns properly identified a dilated aortic root. Seven of the nine (78%) correctly included aortic insufficiency or aortic regurgitation in their differential diagnosis. This was increased compared to the rest of the interns, where only 23 of the 44 (52%) correctly included aortic insufficiency or aortic regurgitation on their differential. All six interns who properly identified the IVC correctly identified that it was collapsible and not dilated. The most common causes for “unsatisfactory” or “borderline” scores in correctly identifying structures were due to errors in obtaining correct views rather than the ability to identify anatomy.

Ten of the 11 interns who obtained the correct views of PLAX and PSAX received satisfactory scores for identifying the correct findings. The one intern who obtained the correct views PLAX and PSAX views but still obtained less than satisfactory scores in their ability to identify findings incorrectly reported right ventricular dilation and a thrombus in the left ventricle.

Interns' physical exam technique was statistically significantly associated with their POCUS exam technique (r=0.46, p=<0.01; Table 3). Identifying physical exam findings was also significantly associated with identifying POCUS findings (r=0.54, P<.01). POCUS technique was significantly associated with identification of correct POCUS findings, formulation of a correct differential diagnosis, and selection of appropriate clinical management (r=0.41, p=<0.01, r=0.60, p=<0.01, r=.32, p= =<0.01 respectively).

**Table 3.** Correlation between an intern's performance in each clinical domain in the Assessment of Examination and Communication Skills (APECS).

	Physical Exam Technique	Identifying Correct Physical Exam Findings	POCUS Exam Technique	Identifying Correct POCUS Findings	Differential Diagnosis	Clinical Management	Maintaining Patient Welfare
**Physical Exam Technique**	1.00000	0.41 *<0.001*	0.46 *<0.001*	0.19 0.129	0.33 *<0.001*	0.23 *0.014*	0.21 *0.025*
**Identifying Correct Physical Exam Findings**		1.00000	0.45 *<0.001*	0.54 *<0.001*	0.43 *<0.001*	0.29 *0.002*	0.16 0.09
**POCUS Exam Technique**			1.00000	0.41 *<0.001*	0.60 *<0.001*	0.32 *<0.001*	0.034 0.789
**Identifying Correct POCUS Findings**				1.00000	0.25 *0.047*	0.32 *0.002*	0.16 0.091
**Differential Diagnosis**					1.00000	0.46 *<0.001*	0.04 0.671
**Clinical Management**						1.00000	0.02 0.833
**Maintaining Patient Welfare**							1.00000

## Discussion

Few studies have objectively assessed the competency of internal medicine trainees in performing a cardiovascular POCUS exam. Our study adds to the growing body of literature by assessing POCUS competency, but also by reporting common mistakes in POCUS technique among internal medicine interns. In a formal assessment of cardiac POCUS skills, most interns lacked the necessary skills to independently perform a cardiac POCUS exam. However, since POCUS technique was significantly associated with both identifying the appropriate findings and including the correct diagnosis on the differential, our results suggest that dedicated teaching of POCUS skills could improve POCUS accuracy and overall diagnostic accuracy in the context of cardiovascular disease. Interns who received satisfactory scores on their POCUS technique were more likely to correctly include aortic insufficiency in their differential diagnosis, further highlighting the effectiveness of POCUS in improving diagnostic accuracy.

The benefits of POCUS are well described. Cardiac POCUS significantly increases the sensitivity and specificity for diagnosing many cardiac abnormalities. For example, among patients with shortness of breath and clinical evidence of volume overload, bedside cardiac POCUS has an 80.6% sensitivity for identifying a reduced ejection fraction [22]. When assessing volume status, the physical exam can be challenging for residents who have little experience visualizing neck veins. Cardiac POCUS assessment of the IVC has a sensitivity of 70% for detecting elevated right atrial pressure [7,23]. Over 75% of internal medicine residents who underwent formal POCUS teaching improved their assessment of systolic function and left ventricular chamber size [6,7]. Since POCUS detects cardiovascular pathology an average of 22 hours earlier than standard echocardiography, incorporating its findings into patient management can expedite and improve care [15].

One major limitation of cardiac POCUS is the variability and inconsistency of technique among users, especially trainees. It remains unclear how many supervised cardiac POCUS exams are needed to achieve competency [16]. The accessibility of hand-held POCUS devices has outpaced opportunities for formalized training and direct observation [11,24,25]. With close to 90% of interns in this study performing below satisfactory in both their POCUS technique and ability to identify the correct findings, there is a risk that trainees will formulate incorrect diagnoses and management plans when using POCUS. For example, improper techniques that were frequently seen within our study, such as incorrect probe orientation or depth, can lead to foreshortening of the left ventricle, failing to visualize pericardial effusions, or incorrectly assessing cardiac function. Lack of direct supervision while performing POCUS exams also limits the opportunity for trainees to learn subtle probe adjustments that may be necessary to optimize their view. Thirty-four (64%) interns were observed using either incorrect depth or gain, two easily correctable errors with the assistance of a supervising preceptor. Obtaining the correct orientation can often be achieved by making minor adjustments to trainees' positioning and ultrasound probe movements. Placing patients in the proper position (e.g., left lateral decubitus for the apical view) may further optimize the chances of obtaining adequate views.

Another key finding in our study was that 26 (49%) interns neglected to attempt the AP4 or SUBC views, and an extremely low number obtained satisfactory views. While this may have been due to the six-minute time constraint, it raises concerns that interns may lack the appropriate knowledge to obtain the correct orientation and positioning for these views. Cardiac POCUS is best used for assessing left ventricular function, the presence of pericardial effusions, and estimating right atrial pressure [8,3,26,27]. However, the assessment of these findings requires multiple cross-sectional views that cannot be obtained solely with the PLAX and PSAX views [8,28,29]. To estimate right atrial pressure, trainees must gain competency in proper SUBC and IVC views. Left ventricular function can easily be miscalculated using a singular view. In a head-to-head comparison to PSAX and AP4, PLAX was regarded as the worst view to assess left ventricular function [30,31]. Apical views were most likely to correctly identify left ventricular abnormalities [27,30]. Furthermore, AP4 and SUBC views provided more accurate estimations of pericardial effusion size compared to parasternal long and short views [31]. Utilizing information obtained from only PLAX and PSAX views may result in incorrect clinical decisions.

Our study is unique in that it provides not only an objective understanding of interns' proficiency with the cardiac POCUS exam, but identifies key correlations between technique and the ability to identify findings. Almost all the interns who received satisfactory scores for technique also received satisfactory scores for correct findings. This suggests that interns who can obtain the correct POCUS views are able to reliably interpret them. For example, all six interns who correctly identified the IVC were able to appropriately state that the patient had a non-dilated IVC with greater than 50% inspiratory collapsibility. Nonetheless, the importance of teaching trainees how to correctly interpret findings remains paramount as one intern mistakenly reported right ventricular dilation and the presence of a left ventricular thrombus despite correctly obtaining the PLAX and PSAX views and receiving satisfactory scores on their technique. Ultrasound technique was also correlated with the formulation of appropriate differential diagnoses. The percentage of interns who correctly identified aortic insufficiency on their differential was considerably higher among those who received satisfactory scores on their ultrasound technique. However, as very few interns obtained satisfactory scores on ultrasound technique, training programs need to place further emphasis on supervised practice of these components of cardiac POCUS.

There was a significant association between accurate findings on cardiovascular physical exam and those gleaned from cardiac POCUS exam, suggesting that these skills are complementary. Just as a history provides context for selecting and interpreting the physical exam, it is likely that the cardiovascular physical exam provides context for performing and interpreting POCUS exams. For example, an intern who appreciates the characteristic diastolic murmur of aortic insufficiency may be more likely to recognize a dilated aortic root on POCUS, since concern for aortic pathology was initially raised on physical exam. A POCUS curriculum that incorporates traditional cardiac physical exam training is likely to be more effective than a program that focuses only on ultrasound technique and interpretation [32]. It is interesting to note that in a prior study of APECS participants, overall physical exam technique and identifying physical exam findings (including data from non-cardiovascular stations) were not associated with cardiac POCUS technique or identifying cardiac POCUS findings [20]. This could reflect the fact that many individuals who are skilled in the cardiac exam also recognize the value of POCUS in making cardiovascular diagnoses.

Our study has limitations. Our sample size was relatively small, including only 53 interns from two internal medicine programs, so results may not be generalizable. The participants were all first-year residents, so performance on APECS may not reflect the overall POCUS abilities of their respective residency programs. It is possible that our results were confounded by prior POCUS training among participants, either during medical school or in other contexts. For example, interns were randomly selected to participate in APECS at variable points during their intern year. It is possible that interns at the end of their first year had higher scores due to informal training received in their first year. However, since we were not assessing the impact of a specific intern-year POCUS curriculum, prior experience should not have impacted the analysis of the relationships between POCUS technique, identifying POCUS findings and generating a differential diagnosis. In a prior analysis of all POCUS APECS stations, POCUS technique and ability to identify the correct findings did not change over the course of the academic year [20].

Faculty preceptors reported variable cardiac physical exam findings across the seven APECS days. POCUS findings remained consistent across all study days. This variability could reflect day-to-day alterations in patient physiology which impacted physical exam findings. It could also reflect heterogeneity in faculty skill in detecting physical exam findings or the increased sensitivity and specificity of POCUS in detecting cardiac abnormalities [33]. Given the stability of POCUS findings across each assessment day, the variability in agreed upon physical exam findings likely did not impact faculty assessment of POCUS skills.

Our study was conducted in a controlled setting where interns were free of any other clinical responsibilities. There is a possibility that interns may spend more or less time and/or perform a more or less thorough cardiac POCUS exam during a real clinical encounter. Interns were not provided any clinical history other than the initial prompt of “shortness of breath.” While this was done to isolate and identify the association between physical exam and POCUS skills, it reduces the generalizability of our study as trainees typically draw from many clinical data points, in addition to physical exam and POCUS findings, when making clinical decisions.

Finally, our study included only a single patient with one cardiovascular diagnosis, possibly limiting generalizability to other diseases. However, observed errors, particularly in POCUS technique (such as incorrect depth or gain), would likely have occurred during the POCUS exam of any patient with cardiovascular disease. The fact that all interns examined the same patient likely minimized confounding factors that could be present when interns' performance is compared across several patients with varying exam findings and diagnoses.

## Conclusion

Internal medicine interns demonstrated variable proficiency in performing a cardiovascular-focused POCUS exam. Our study provides insights on common errors among trainees when performing a cardiac POCUS exam. Performance on the cardiovascular physical exam was significantly correlated to performance on the cardiac POCUS exam, providing evidence that these skills are complementary. These findings can inform efforts at both the undergraduate and graduate medical level to improve both cardiac physical exam and POCUS skills. Further emphasis on integrated training in cardiac physical exam and POCUS exam techniques would likely lead to increased identification of relevant cardiovascular findings and improve patient care.
